# Association between BNP and all-cause mortality in critically ill children: a cohort study

**DOI:** 10.1038/s41390-024-03666-7

**Published:** 2024-10-23

**Authors:** Zhen Zhang, Yuna Li, Chunfeng Yang, Yumei Li

**Affiliations:** Department of PICU, Children’s Medical Center, The First Hospital of Jilin University, Changchun, China

## Abstract

**Background:**

There is evidence that a high level of BNP is associated with poorer outcomes in patients with cardiac diseases, but few data are available concerning BNP and all-cause mortality in pediatric population.

**Methods:**

Using the 2010–2018 pediatric intensive care database, we conducted a retrospective study on patients aged 28 days to 18 years, analyzing post-admission BNP measurements. Through two-piecewise regression to identify inflection points, and multivariable logistic regression, we investigated BNP’s association with all-cause mortality. We also developed a multivariable-adjusted restricted cubic spline model to explore BNP’s non-linear correlation with mortality.

**Results:**

In a study of 3220 patients, the overall all-cause mortality rate was 6.7%, with rates across BNP quartiles (Q1–Q4) significantly differing, highlighting a notable increase in mortality at higher BNP levels (*P* < 0.001). Specifically, patients with BNP ≥ 10,170 pg/ml had an adjusted mortality odds ratio (OR) of 2.017 (95% CI 1.265–3.217; *P* = 0.0032). Analysis confirmed a non-linear relationship between BNP levels and mortality, with log2 BNP associated with increased risk (OR1.28, 95% CI 1.19–1.38; *P* < 0.001). Subgroup analyses further revealed that very high BNP levels, especially in infants, with lactate ≥2.0 mmol/L, or CKMB ≥ 45 μ/L.

**Conclusions:**

BNP level was associated with all-cause mortality, especially for the patients with BNP ≥ 10,170 pg/ml.

**Impact:**

- This study explored the non-linear association between BNP levels and all-cause mortality in the PICU, finding a significant association among patients with BNP levels above 10,170 pg/ml.
- The study revealed that higher BNP levels are associated with increased mortality in critically ill children, including those with non-cardiac diseases.
- This research provides new data on a Southern Chinese population, previously unstudied, enriching the existing body of knowledge.
- While most studies have focused on adult cardiac patients, this research highlights the importance of BNP as a prognostic tool in the PICU, including non-cardiac cases, adding to the literature.
- This study furnishes novel clinical evidence supporting the monitoring of BNP concentrations within the PICU, aiding in prognostic predictions and the development of tailored treatment plans for patients.

## Introduction

Pediatric Intensive Care Unit (PICU) is a specialized health care unit dedicated to the intensive care of critically ill children. The PICU’s mission is to reduce the mortality rate of critically ill children through intensive monitoring and treatment. Advances in pediatric intensive care have significantly reduced child mortality worldwide, but there are still differences in children’s chances of survival in different parts of the world.^1–3^ China has made more progress in pediatric intensive care in recent decades. However, the overall mortality rate in PICUs is still higher than in developed countries. The majority of resources in PICU are consumed by high-mortality patients. Thus, the need to reduce child mortality remains urgent. Therefore, for early detection and timely treatment of children at high risk of death, it is essential to identify high-mortality patients and to find appropriate biomarkers that predict mortality.

B-type Natriuretic Peptide (BNP) as a clinical biomarker in the assessment of heart failure (HF) is known and can be a significant factor in the diagnosis and treatment.^4,5^ Even in patients without HF, BNP levels are a stronger predictor of death than traditional risk factors.^6^ AHA 2022 AHA/ACC/HFSA guidelines^7^ recommend the use of BNP as a biomarker for the prevention, initial diagnosis, and risk stratification of heart failure. However, the guideline does not mention whether this recommendation can be applied to children and there is no standardized reference cut-off value to children yet. Moreover, elevated BNP levels have been reported not only in patients with cardiac.^8–10^ but also in noncardiac causes,^11–13^ but evidence for its use in noncardiac diseases is rarer. This suggests that further clinical studies and evidence are needed. In adults, several studies have been reported based on small sample sizes.^14,15^ However, to our knowledge, no study has assessed the association between BNP and all-cause mortality in pediatric population. To bridge this knowledge gap, we assess the relationship between BNP and prognosis of critically ill children in China using data from the pediatric intensive care (PIC) database.

## Methods

### Study design and participants

A population-based cohort study was conducted using the PIC database (version 1.1.0), which was a large China-based pediatric critical care database. PIC database included 13,499 ICU stays between 2010 and 2018. Zhang Z obtained approval to access this database. The data has been previously deidentified, and the institutional review boards of the Massachusetts Institute of Technology approved the use of the database for research. We have also complied with all relevant ethical regulations regarding the use of the data for our study.

Individuals aged ≤28 days or >18 years, and individuals with missing BNP data were not included in this study. Even though some patients were recurrently admitted to PICU, we considered only the first hospital and first PICU admission. Data of patients on second or more PICU admissions would be excluded. The final cohort contained 3220 patients.

### Main exposure

The primary independent variable was BNP. Only the first BNP value of each patient was used in this study.

### Covariates

Patient characteristics that were previously shown to explain most of the variation in mortality. The following variables were included in our study: registered information of admission (sex, age), vital signs, and laboratory tests, such as lactate, creatine kinase-MB (CKMB), albumin, blood urea nitrogen (BUN), creatinine, alanine aminotransferase (ALT), glucose, hemoglobin, white blood cell (WBC), C-reactive protein (CRP). Comorbidities (heart disease, respiratory diseases, infection, tumors, accidents deformities and others) were also recorded. The worst values of vital signs and laboratory tests on the first day were taken. The laboratory data in this database are all sourced from the clinical laboratories of a tertiary hospital, ensuring the reliability and high quality of the data. All covariates had missing values of 5% or less. These data were excluded from the statistical analysis.

### End point

The primary endpoint of this study is all-cause mortality. The timing of the primary end point was defined as time to death in PICU but did not include death after discharge from PICU.

### Statistical analysis

Descriptive analysis was performed for all participants. Data are expressed as mean ± standard deviation (SD) or median (interquartile range) for continuous variables, and as frequency or percentage for categorical variables. For baseline characteristics analysis, continuous variables are compared using *t*-tests or rank-sum tests, while categorical variables are assessed using *χ*2 tests. Multivariable logistic regression models were built to adjust for potential confounders in the association between the BNP and all-cause mortality, which were shown as odds ratios (ORs) with 95% confidence intervals (CIs). Both non-adjusted and multivariate-adjusted models were used. Model I was adjusted for sex and age; Model II was adjusted for sex, age, and disease categories; and Model III was adjusted for sex, age, disease categories, lactate, albumin, CKMB, BUN, Creatinine, ALT, CRP, and Hemoglobin. Additionally, sensitivity analysis was performed to improve the robustness of the results. BNP was used as a categorical variable in the logistic regression models and a trend test was performed. A multivariate-adjusted restricted cubic spline model was constructed to establish the OR curves at 4 knots to examine the possible nonlinear dose-response association between BNP and all-cause mortality. The cut-off value was determined by logistic inflection point analysis. A two-piecewise logistic regression model was developed to assess the relationship between BNP and all-cause mortality, with adjustment for potential confounders included in model III. Analyses stratified according to clinical significance and the result of univariate analysis (*P* < 0.05), including sex, age, heart disease, Lac, CKMB and CRP, examine the effect of these factors on the above associations. Interactions across subgroups were tested using the likelihood ratio test.

All analyses were performed using the statistical software packages R 4.0.2 (http://www.R-project.org, The R Foundation) and Free Statistics software versions 1.8. A two-tailed test was performed and *P* < 0.05 was considered statistically significant.

### Statement

This study was approved by the Ethics Committee of the First Hospital of Jilin University on March 5, 2024.

## Results

### Characteristics of the cohort

**Fig. 1:**
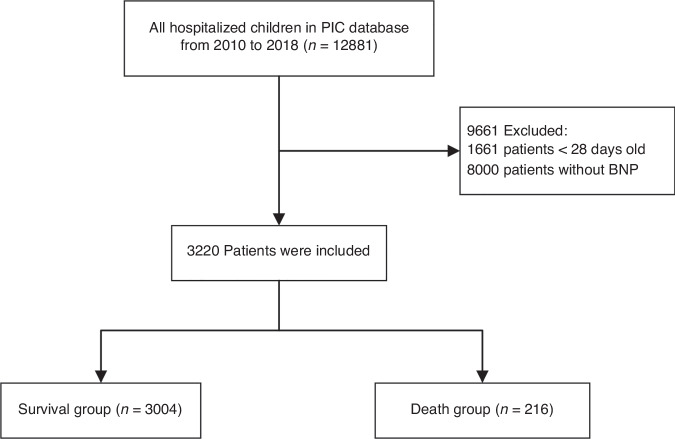
Flow chart.

Of 12,881 pediatric patients with first hospital and ICU admission in PIC from 2010 to 2018, The final cohort contained 3220 patients after exclusion. Among them, 3004 patients who survived until discharge from the hospital were defined as survivors, and 216 patients (6.7%) who died in the hospital were included in the death group (Fig. 1). Baseline characteristics of all patients according to BNP categories are summarized in Table 1. The median age of the study participants was 1.2 years(range 0.4–3.3 years), and 1540 (47.8%) patients were female. In addition, there were 1998 (62.0%) patients with heart disease. The rest were classified as respiratory diseases, infections, tumors, accidents, deformities and others. A higher BNP level was associated with a lower age (0.4 versus 3.0), higher heart rate (145.8 ± 23.4 versus 133.2 ± 26.4), higher lactate (2.4 versus 1.7), higher CKMB (60.0 versus 31.0). And all-cause mortality increased with increasing BNP levels (107[13.3%] versus 39[4.8%]).

**Table 1:** Baseline patient characteristics according to BNP level in the PIC database.

Variables	Total (*n* = 3220)	BNP ≤685 pg/ml (*n* = 805)	BNP 687~2647 pg/ml (*n* = 805)	BNP 2650~7637 pg/ml (*n* = 805)	BNP ≥7639 pg/ml (*n* = 805)	*p*
Sex, *n* (%)						0.066
Female	1540 (47.8)	363 (45.1)	412 (51.2)	393 (48.8)	372 (46.2)	
Male	1680 (52.2)	442 (54.9)	393 (48.8)	412 (51.2)	433 (53.8)	
Age(years)	1.2 (0.4, 3.3)	3.0 (1.2, 6.7)	2.0 (0.9, 4.2)	0.8 (0.4, 1.7)	0.4 (0.2, 0.8)	<0.001
All-cause mortality, *n* (%)						<0.001
No	3004 (93.3)	766 (95.2)	771 (95.8)	769 (95.5)	698 (86.7)	
Yes	216 (6.7)	39 (4.8)	34 (4.2)	36 (4.5)	107 (13.3)	
Disease categories, *n* (%)						<0.001
Heart disease	1998 (62.0)	330 (41)	552 (68.6)	603 (74.9)	513 (63.7)	
Respiratory diseases	485 (15.1)	111 (13.8)	91 (11.3)	110 (13.7)	173 (21.5)	
Sepsis and non-respiratory infections	122 (3.8)	45 (5.6)	23 (2.9)	25 (3.1)	29 (3.6)	
Tumor	149 (4.6)	73 (9.1)	36 (4.5)	18 (2.2)	22 (2.7)	
Accidents (including trauma and poisoning)	192 (6.0)	129 (16)	34 (4.2)	13 (1.6)	16 (2)	
Deformities and others	274 (8.5)	117 (14.5)	69 (8.6)	36 (4.5)	52 (6.5)	
Heart rate,(beats/min)	133.2 ± 26.4	120.5 ± 28.9	128.1 ± 23.8	138.5 ± 21.9	145.8 ± 23.4	<0.001
Lac,(mmol/L)	1.9 (1.3, 2.8)	1.7 (1.2, 2.6)	1.8 (1.3, 2.7)	1.9 (1.4, 2.5)	2.4 (1.6, 3.5)	<0.001
CKMB,(μ/L)	45.0 (29.0, 66.0)	31.0 (21.0, 55.0)	39.0 (28.0, 57.5)	49.0 (36.0, 65.0)	60.0 (40.0, 85.0)	<0.001
ALB,(g/L)	38.6 ± 5.3	39.1 ± 5.7	38.8 ± 5.0	38.9 ± 4.5	37.6 ± 5.6	<0.001
BUN,(mmol/L)	4.2 ± 3.4	3.9 ± 2.1	4.0 ± 3.8	3.9 ± 2.3	5.0 ± 4.6	<0.001
Crea,(umol/L)	40.0 (33.0, 48.6)	41.2 (34.0, 50.0)	38.0 (31.0, 46.0)	38.0 (31.0, 44.0)	44.0 (35.0, 55.8)	<0.001
ALT,(μ/L)	21.0 (14.0, 33.0)	19.0 (13.0, 32.0)	20.0 (13.0, 30.0)	22.0 (15.0, 32.0)	25.0 (17.0, 38.0)	<0.001
Glu,(mmol/L)	9.1 ± 4.4	8.3 ± 4.8	8.6 ± 3.6	9.5 ± 3.9	10.1 ± 4.9	<0.001
Hb,(g/L)	105.8 ± 19.8	112.7 ± 20.7	103.2 ± 17.4	102.6 ± 16.9	104.5 ± 21.8	<0.001
WBC,(×10^**9**^/L)	9.4 (6.6, 13.4)	10.0 (7.0, 14.3)	10.2 (7.3, 14.4)	9.0 (6.4, 12.8)	8.2 (5.9, 12.1)	<0.001
CRP,(mg/L)	28.3 (4.0, 56.0)	5.0 (0.1, 25.8)	34.0 (5.0, 63.0)	42.7 (19.0, 71.0)	30.2 (4.2, 55.6)	<0.001

Data are expressed as mean ± standard deviation, median (interquartile range), or *n* (%).

*BNP* B-type natriuretic peptide, *Lac* lactate, *CKMB* creatine kinase-MB, *ALB* albumin, *BUN* blood urea nitrogen, *Crea* creatinine, *ALT* alanine aminotransferase, *Glu* glucose, *Hb* hemoglobin, *WBC* white blood cells, *CRP* C-reactive protein.

### Association between BNP and all-cause mortality

**Fig. 2:**
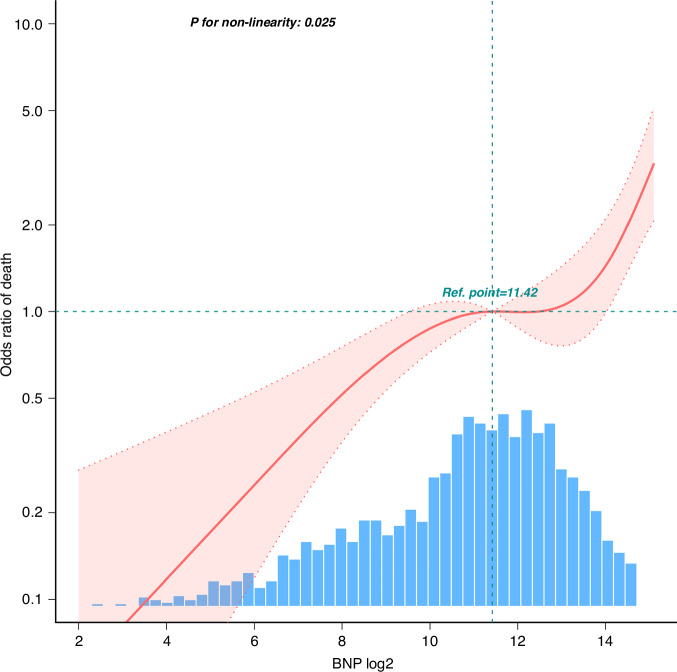
Spline plot for the associations of BNP with all-cause mortality. The restricted cubic spline model was adjusted for sex+age+disease categories+ALB+BUN+CRP+Hb+Lac+log ALT+log Crea+log CKMB. BNP B-type natriuretic peptide, Lac lactate, CKMB creatine kinase-MB, ALB albumin, BUN blood urea nitrogen, Crea creatinine, ALT alanine aminotransferase, Hb hemoglobin, CRP C-reactive protein.

By applying the two-piecewise regression models, we identified the critical turning point at BNP = 10,170. This segmented modeling approach allows us to more finely capture changes in the data, enhancing the model’s ability to fit complex relationships. In the two-piecewise regression models, the adjusted OR of all-cause mortality was 2.017 (95% CI, 1.265–3.217; *P* = 0.0032) in participants with BNP ≥ 10,170 pg/ml, whereas there was no association between BNP and all-cause mortality in participants with BNP < 10,170 pg/ml (Table 2). TheORs and corresponding 95% CIs for the risk for all-cause mortality according to BNP [Log2], BNP ≥ 10,170 pg/ml and BNP quantiles are summarized in Table. 3. A high BNP [log2] was associated with an increased all-cause mortality (OR 1.28, 95% CI 1.19–1.38; *P* < 0.001), after adjusting for sex, age, disease categories, lactate, albumin, CKMB, BUN, creatinine, ALT, CRP and hemoglobin. Similar results were obtained after subgrouping based on an BNP level of 10,170 pg/ml (OR 2.96, 95% CI 2.06–4.24; *P* < 0.001). The association was maintained when the BNP level was transformed into a categorical variable. Compared with the lowest BNP group Q1(≤685 pg/ml), the adjusted OR values for BNP and mortality in Q2(687~2647 pg/ml), Q3(2650~7637 pg/ml), and Q4(≥7639 pg/ml) were 2.1(95% CI 1.2–3.67, *P* = 0.009), 2.48(95% CI 1.39–4.43, *P* = 0.002), and 4.68 (95% CI 2.75–7.98, *P* < 0.001), respectively. Accordingly, the association between BNP and all-cause mortality was nonlinear (*P* = 0.025) in the restricted cubic spline model (Fig. 2).

**Table 2:** Association between BNP and all-cause mortality using two-piecewise regression models.

BNP	Crude model		Adjusted model	
	OR(95%CI)	*P*-value	OR(95%CI)	*P*-value
<10,170 pg/ml	0.951 (0.86~1.051)	0.3223	1.074 (0.963~1.198)	0.2019
≥10,170 pg/ml	2.42 (1.859~3.151)	<0.001	2.017 (1.265~3.217)	0.0032

Adjusted for sex+age+disease categories+Lac+ALB+BUN+CRP+Hb+log ALT+log Crea+log CKMB.

*BNP* B-type natriuretic peptide, *Lac* lactate, *CKMB* creatine kinase-MB, *ALB* albumin, *BUN* blood urea nitrogen, *Crea* creatinine, *ALT* alanine aminotransferase, *Hb* hemoglobin, *CRP* C-reactive protein.

**Table 3:** Association between BNP and all-cause mortality in the PIC database.

Variable	No-adjusted model		Model I		Model II		Model III	
	OR(95%CI)	*P*-value	OR(95%CI)	*P*-value	OR(95%CI)	*P*-value	OR(95%CI)	*P*-value
log BNP	1.25 (1.17~1.33)	<0.001	1.3 (1.21~1.39)	<0.001	1.36 (1.27~1.45)	<0.001	1.28 (1.19~1.38)	<0.001
Binary variable								
BNP < 10,170 pg/ml	Ref.		Ref.		Ref.		Ref.	
BNP ≥ 10,170 pg/ml	3.62 (2.72~4.8)	<0.001	3.97 (2.94~5.35)	<0.001	4.21 (3.09~5.73)	<0.001	2.96 (2.06~4.24)	<0.001
BNP quantile								
Q1(≤685 pg/ml)	Ref.		Ref.		Ref.		Ref.	
Q2(687~2647 pg/ml)	0.87 (0.54~1.39)	0.549	0.96 (0.6~1.55)	0.882	1.48 (0.9~2.44)	0.12	2.1 (1.2~3.67)	0.009
Q3(2650~7637 pg/ml)	0.92 (0.58~1.46)	0.723	1.12 (0.69~1.82)	0.652	1.91 (1.14~3.19)	0.014	2.48 (1.39~4.43)	0.002
Q4(≥7639 pg/ml)	3.01 (2.06~4.41)	<0.001	3.75 (2.47~5.69)	<0.001	5.75 (3.67~9)	<0.001	4.68 (2.75~7.98)	<0.001

Model I: Adjusted for sex + age;

Model II: adjusted for Model I + disease categories;

Model III: adjusted for Model II+Lac+ALB+BUN+CRP+Hb+log ALT+log Crea+log CKMB.

*BNP* B-type natriuretic peptide, *Lac* lactate, *CKMB* creatine kinase-MB, *ALB* albumin, *BUN* blood urea nitrogen, *Crea* creatinine, *ALT* alanine aminotransferase, *Hb* hemoglobin, *CRP* C-reactive protein.

### Subgroup analysis

**Fig. 3:**
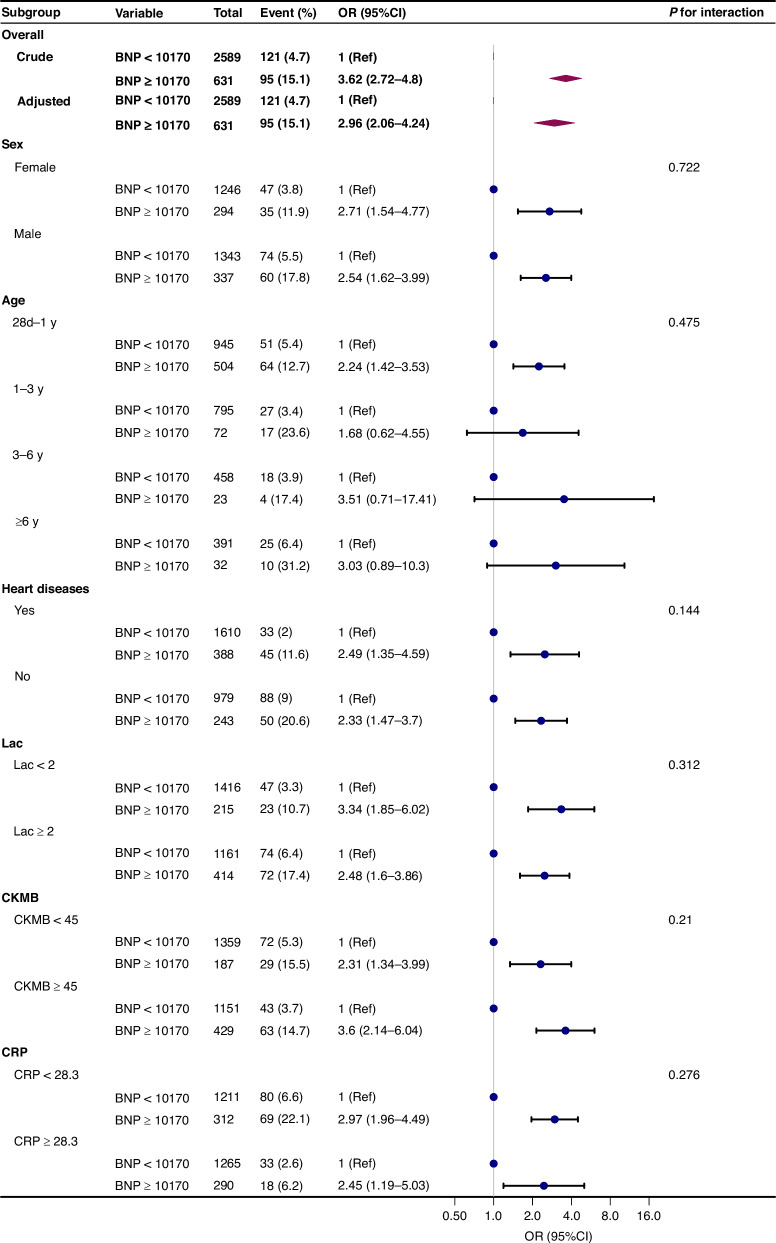
Stratified multivariable analysis of the association between BNP and all-cause mortality according to baseline characteristics. Notes: Each stratification adjusts for all factors (sex, age, Lac, ALB, BUN, Hb, log CKMB, log ALT, log Crea) except for the stratification factor itself. BNP B-type natriuretic peptide, Lac lactate, CKMB creatine kinase-MB, ALB albumin, BUN blood urea nitrogen, Crea creatinine, ALT alanine aminotransferase, Hb hemoglobin, CRP C-reactive protein.

To detect whether the association between BNP levels and all-cause mortality was present in different subgroups,analyses and interactive analyses were stratified according to clinical significance and the result of univariate analysis (if *P* < 0.005, selected) (Fig. 3). These stratification criteria for age, lactate, CK-MB, and CRP are derived from the extensive experience accumulated by medical professionals in clinical practice. This stratification not only takes into account the variations of these biomarkers across different age groups and physiological states but also integrates their performances in specific disease contexts. No variable played an interactive role in the association between BNP and all-cause mortality (*P* for interaction >0.05). Nevertheless, in the BNP ≥ 10,170 pg/ml subgroup, with age ≤1 years, Lac ≥2.0 mmol/L and CKMB ≥ 45 μ/L were associated with a greater risk for all-cause mortality compared with the corresponding subgroup. Subgroup analyses were adjusted for sex, age, Lac, ALB, BUN, creatinine, Hb, CKMB [log2], ALT [log2], creatinine [log2] (Fig. 3).

## Discussion

Early identification of the risk of death in critically ill children can help to accurately stratify different critically ill children for different monitoring and management measures. This study’s subgroup analyses suggest that BNP levels are useful in identifying critically ill children at high risk of death. This representative cohort study demonstrates a non-linear association between BNP level and all-cause mortality. The association remained robust in sensitivity and subgroup analyses.

In some adults’ studies, BNP is a strong predictor of death in patients in a clinical setting, whether HF is present or not.^6,16^ Some clinically-based studies found that higher levels of BNP is associated with a greater risk for adverse outcomes in patients with HF, including all-cause and cardiovascular death and major cardiovascular events.^13,17^ A study from Copenhagen measured pro-atrial natriuretic peptide and N-terminal pro-BNP in stored blood samples from 1337 consecutive patients hospitalized acutely for any cause in 1998–1999.^12^ Compared with the lowest quartile, higher quartiles of natriuretic peptide levels were associated with increased risk of death in the overall population and in the subgroup without known cardiovascular disease. In contrast to previous studies, the present study analyzed data from PIC after adjusting for potential confounders using multivariate regression analysis. This allows the results to be generalizable to a larger group of children. The results of the restricted cubic spline model showed a nonlinear relationship between BNP and all-cause mortality in critically ill children. Among logistic inflection point analysis, the risk of death in critically ill children did not increase with increasing BNP in individuals with BNP < 10,170 pg/ml, whereas the risk of death increased with increasing BNP in individuals with BNP ≥ 10,170 pg/ml; in other words, the risk of death in critically ill children increased only when BNP levels reached a certain level. Furthermore, Clinical studies have shown a significant correlation between BNP levels and the occurrence of heart disease,^18–20^ and this association has also been observed in the present study.

In Chinese pediatric HF guidelines,^21^ BNP was recommended for diagnosis and assessment of prognosis, but unfortunately no standardized risk thresholds were given and no recommendations were given in non-heart failure children. Our study focused on a Chinese pediatric population and can complement previous work. The PIC data were obtained from the Children’s Hospital of Zhejiang University School of Medicine in China, which is one of the top 5 pediatric medical centers in China and receives children from most of the southern part of the country. Therefore, our results should be applicable to most of the pediatric population in southern China.

Elevated levels of both BNP and NT-proBNP have been reported in patients with a variety of cardiac and noncardiac causes, including advancing age, anemia, renal failure, obstructive sleep apnea, severe pneumonia, pulmonary embolism, pulmonary arterial hypertension, critical illness, bacterial sepsis, and COVID-19 infection.^22–26^ Michelle reported that plasma BNP levels were a stronger risk factor than traditional risk factors in patients without HF. The risk of death associated with BNP level was similar between patients with and without HF, especially in the acute care setting.^13,14,20,27^

In children, previous studies have focused on cardiac diseases such as heart failure, congenital heart disease, and Kawasaki disease in children, but rare studies have investigated the relationship between BNP and non-cardiac diseases.^10,28,29^ Our subgroup analysis showed that high BNP levels remain associated with all-cause mortality even in critically ill children with noncardiac diseases. Due to the limited sample size of patients with BNP and non-cardiac diseases in this study, this result should be interpreted with caution and more well-designed prospective studies in this area are needed.

## Limitation

The present study has several limitations. First, although the large sample size of our study minimized sampling error, the fact that it was a single-center retrospective study does not represent the generalizability of the findings to other health care settings. Second, although the findings raise questions about the potential risk of all-cause mortality, the interpretation of the results is limited by the observational nature of the study, and we are unable to draw any causal inferences. Third, this was a retrospective study, and the data on deaths may be biased by the fact that only in-hospital mortality was available and post-discharge follow-up was missing. Future studies should include larger multicenter trials to better reflect real-world outcomes.

## Conclusion

The results of this cohort study suggest a nonlinear association between BNP levels and all-cause mortality in critically ill children in China. Therefore, the monitoring and management of critically ill children should consider the plasma BNP level in assessing high risk of mortality.

## Data Availability

The data that support the findings of this study are available from the pediatric intensive care (PIC) database but restrictions apply to the availability of these data, which were used under license for the current study, and so are not publicly available. Only by applying through the *physionet.org* website can one obtain permission to download the data.
